# Self-Assembled Antimicrobial Nanomaterials

**DOI:** 10.3390/ijerph15071408

**Published:** 2018-07-04

**Authors:** Ana Maria Carmona-Ribeiro

**Affiliations:** Biocolloids Laboratory, Instituto de Química, Universidade de São Paulo; Av. Prof. Lineu Prestes 748, São Paulo 05508-000, Brazil; amcr@usp.br; Tel.: +55-011-3091-1887

**Keywords:** antimicrobial lipid bilayers, bilayer fragments with microbicides, assemblies with antimicrobial polymers and peptides, disassembly upon interaction with microbes, antimicrobial hybrid nanoparticles and thin nanostructured films

## Abstract

Nanotechnology came to stay improving the quality of human life by reducing environmental contamination of earth and water with pathogens. This review discusses how self-assembled antimicrobial nanomaterials can contribute to maintain humans, their water and their environment inside safe boundaries to human life even though some of these nanomaterials display an overt toxicity. At the core of their strategic use, the self-assembled antimicrobial nanomaterials exhibit optimal and biomimetic organization leading to activity at low doses of their toxic components. Antimicrobial bilayer fragments, bilayer-covered or multilayered nanoparticles, functionalized inorganic or organic polymeric materials, coatings and hydrogels disclose their potential for environmental and public health applications in this review.

## 1. Introduction

The assembly of similar or different molecules driven by intermolecular interactions is ubiquitous in Nature and can be a source of inspiration for the construction of novel and effective bioactive supramolecular assemblies (BSA) [1,2,3]. Most warranted, the antimicrobial and hybrid self-assembled nanomaterials open new avenues to solve major resistance problems created by the continuous use of antibiotics, antimicrobials and disinfectants [4]. In combination, active molecule(s) and nanomaterials may assume a variety of shapes [5,6] such as nanoparticles [6,7,8,9,10,11,12,13,14,15,16,17,18,19,20,21,22,23,24], nanodisks [25,26,27], films [28,29,30], coatings [31,32,33], and hydrogels [30,34,35,36,37,38]. The active molecules they carry can be the antimicrobial lipids [28,29,39], surfactant [40,41], polymer [42], or peptide [19,20,25,27,30,43,44], the metallic or metal oxide nanoparticles [45,46], nanotubes [30,43,47,48], and antibiotics or drugs [4,6,21]. The inherent advantages of BSA are twofold: (1) facile combination of different antimicrobials with nanomaterials; (2) facile disassembly in front of the pathogen. Furthermore, nanomaterials often display a very useful antimicrobial activity by themselves. Therefore, the combinations between nanomaterials and a variety of antimicrobials represent an important field for multidisciplinary research. The intermolecular interactions between the carrier and the active molecule(s) keep the molecules of the supramolecular assembly together for subsequent disassembly upon contact with the pathogen and cargo release [3,39]. In this review, BSA with lipids, metals and metal oxide nanoparticles, antibiotics, polymers, and peptides reveal the ever-increasing importance of combinations for fighting the ever-increasing resistance of microbes to antimicrobials. 

## 2. BSA with Lipids

Natural lipids as the phospholipids are in general inert against microbes but they have been recognized as important matrixes that provide appropriate microenvironments for a variety of bioactive small or large molecules with especial applications in drug and vaccine delivery. Similarly, the cationic synthetic lipid dioctadecyldimethylammonium bromide (DODAB) assembled to yield bilayers shaped as bilayer nanodisks, vesicles, or coatings with polymers has been revealing not only a fundamental scaffold utility for supporting active molecules but also an interesting activity against microbes due to quaternary nitrogen on its positively charged headgroup. 

**Figure 1:**
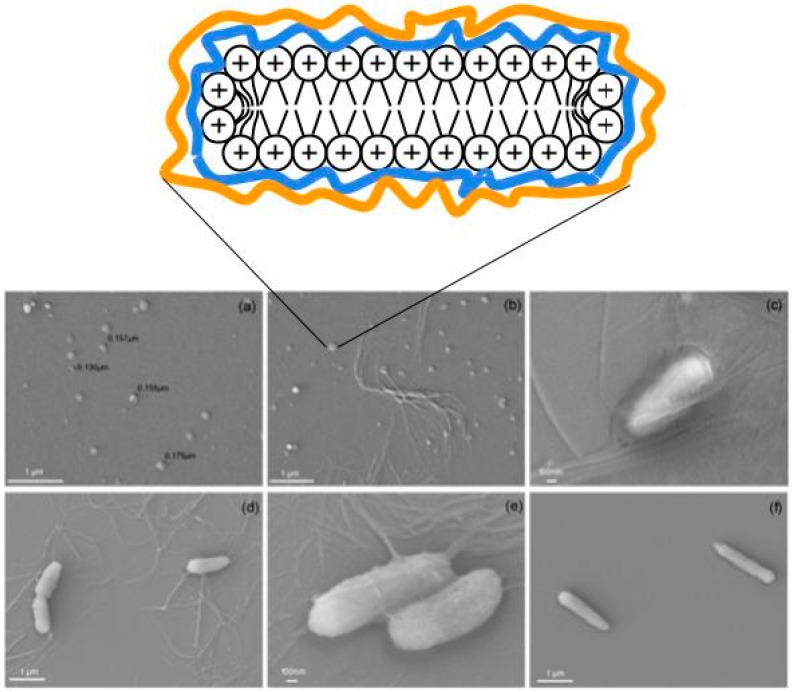
The interaction between *Pseudomonas aeruginosa* multidrug resistant (MDR) cells and bioactive supramolecular assemblies (BSA) made of dioctadecyldimethylammonium bilayer (DODAB) nanodisks surrounded by carboxymethylcellulose (CMC) in blue and poly (diallyl dimethyl ammonium) chloride (PDDA) in orange. (**a**) Scanning electron microscopy SEM ) image of BSA. (**b**) SEM image of BSA and *P. aeruginosa* MDR cells at 4.4 × 10^3^ cells/mL. (**c**) SEM image of BSA and *P. aeruginosa* MDR cells at 8.9 × 10^5^ cells/mL (**d**), and (**e**) SEM images of PDDA (1.0 mg/mL) and *P. aeruginosa* MDR cells at 8.9 × 10^5^ cells/mL. (**f**) Control of *P. aeruginosa* MDR cells only. Final concentrations in the BSA are 0.05 mM DODAB, 0.05 mg/mL CMC and 0.05 mg/mL PDDA. The cross-section scheme for DODAB nanodisk/CMC/PDDA was adapted from references [7] and [9]. Scheme for the nanostructure adapted with permission from reference [9]. Copyright (2010) American Chemical Society. Photos reproduced from reference [5].

Figure 1 illustrates the use of DODAB bilayer nanodisks as scaffolds to support polymer layers of anionic carboxymethylcellulose (CMC) and the cationic, nonhemolytic antimicrobial polymer poly (diallyl dimethyl ammonium) chloride (PDDA) [26]. The disassembly of PDDA, CMC and DODAB from the BSA and assembly with the cell wall of a multidrug-resistant (MDR) bacterial strain provided effective bactericidal action [5] (Figure 1). Damages to the bacterial cells involved disruption of the cell wall and cell membrane with leakage of bacterial cell contents and appearance of multilayered fibers made of the BSA components and the biopolymers withdrawn from the cells. Figure 1a shows the cross section of the BSA constructed with DODAB bilayer fragments (DODAB BF) or nanodisks surrounded by the anionic biopolymer carboximethylcellulose (CMC) in blue and the cationic antimicrobial polymer PDDA in orange. These nanostructures caused the disassembly of the biopolymers of the cell wall of *Pseudomonas aeruginosa* as seen from the scanning electron micrographs (SEM) of the bacterial cells in the presence of the DODAB BF/CMC/PDDA disks (Figure 1b–d) [5,9]. Other cationic antimicrobial polymers and their assemblies were also reported to act against microorganisms via similar mechanisms [42,49].

**Figure 2:**
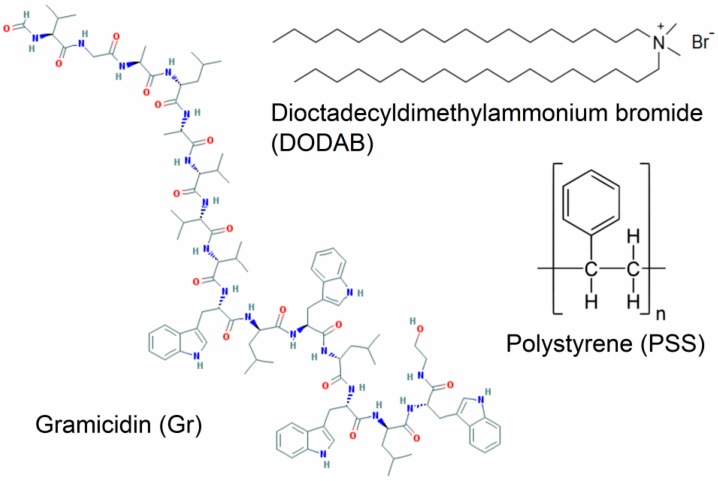
Chemical structure of the antimicrobial peptide Gr, the antimicrobial lipid DODAB, and the polymer polystyrene sulfate (PSS) used to build the antimicrobial BSA shown on Figure 3. The sulfate moiety in PSS is attached to the polystyrene chain terminus due to the polymerization of styrene in the presence of potassium persulfate as initiator.

**Figure 3:**
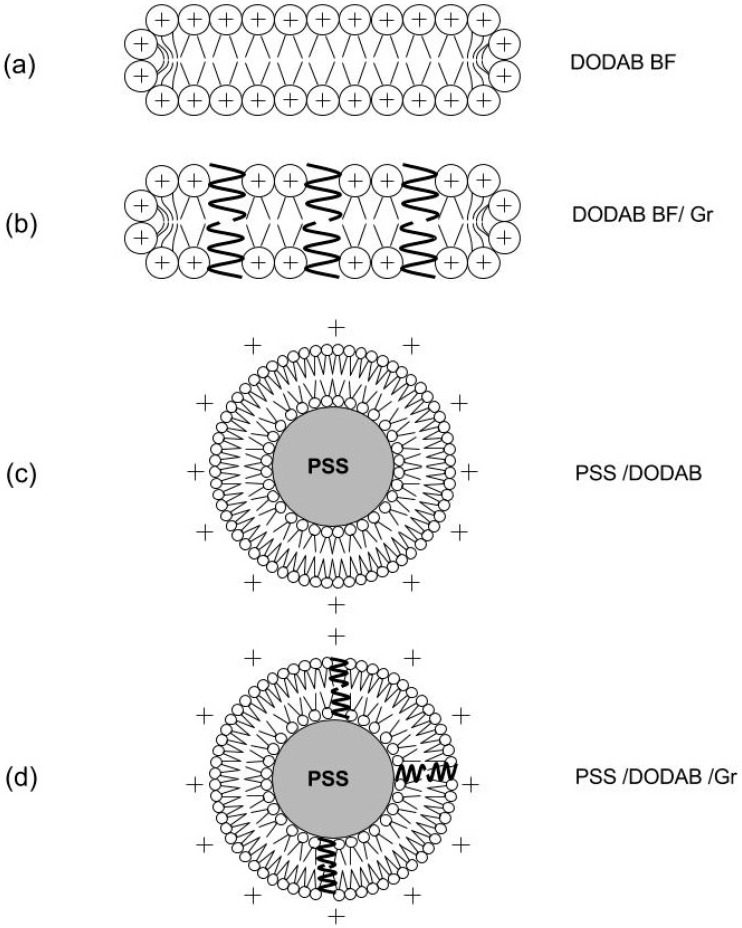
Some cationic BSA with two antimicrobials: the cationic lipid DODAB and the antimicrobial and neutral antimicrobial peptide Gr. (**a**) Bilayer fragments (BF) of the cationic lipid DODAB [26,39,52]; (**b**) BF incorporating dimeric channels of Gr [11,59]; (**c**) PSS NPs covered by a DODAB bilayer [12,65,66,67]; and (**d**) PSS NPs covered by a DODAB bilayer with inserted Gr dimeric channels [10]. Schematic representations are cross-sections. Reproduced from [10].

The second important property of the cationic lipid DODAB [39,50,51,52] self-assembled as vesicles or bilayer fragments (BF or nanodisks) is its antimicrobial activity [53,54,55,56]. This was intensively and extensively explored over the last two decades by itself or in combination with a variety of other antimicrobials such as amphotericin B [7,56,57], rifampicin [8], gramicidin D [10,11,58,59], clarithromycin [6] or the antimicrobial polymer PDDA [7,9,28]. Combinations between DODAB and biocompatible polymers such as acrylates films [29] or poly (methyl methacrylate) (PMMA) nanoparticles [60], or carboxymethylcellulose [7,9,61] also yielded adequate antimicrobial formulations. For example, DODAB bilayer fragments (BF) or nanodisks solubilized amphotericin B in aqueous dispersions and yielded a low cost formulation very effective in vivo in a mice model of systemic candidiasis which was active in absence of nephrotoxicity [56,57,62]. Alternatively, DODAB effectively covered amphotericin B aggregates yielding the DODAB-covered nanoparticles of amphotericin B [63]. More recently, DODAB BF combined with the antimicrobial peptide gramicidin D yielded broad-spectrum microbicidal formulations with the peptide dimers at the borders of the BF [58,59] or embedded in the DODAB cationic bilayer as functional dimeric channels able to affect the ionic balance of the bacterial cells thereby exerting their bactericidal action [11]. In addition, polystyrene sulfate nanoparticles covered with single DODAB bilayers [12,13,64,65] also proved useful to insertion of gramicidin D reconstituting the functional dimeric channel of gramicidin D and yielding broad microbicidal effect [10]. Figure 2 shows the chemical structures of gramicidin D (Gr), DODAB and polystyrene further assembled in Figure 3 to yield some microbicidal combinations as the DODAB BF, DODAB BF/Gr, PSS/DODAB and PSS/DODAB/Gr. 

Although amphotericin B formulation in cationic lipid nanodisks effectively treated candidiasis in a mice model in virtual absence of nephrotoxicity [57,62], the high dose of the cationic lipid in the formulation was toxic for the liver [68]. This effective and non-nephrotoxic amphotericin B (AMB) formulation with DODAB BF (named DODAB/AMB) led to a toxicity survey in mice at low drug to lipid molar ratios where hepatotoxicity, spleen damage and blood changes in comparison to DOC/AMB (sodium deoxy-cholate/amphotericin B, Fungizone) showed toxic effects associated to DODAB only [68]. Swiss Webster female mice were given DODAB, DODAB/AMB or DOC/AMB intraperitonially (ip) for 10 consecutive days (0.4 mg/kg/day AMB; 80 mg/kg/day DODAB). Repeated dose-toxicity was evaluated at the end of the treatment period (on day 11) and after a recovery period of six months from biochemical and hematological parameters plus histopathological examination of the spleen and liver both at days 11 and 180. DODAB in the formulation administered intraperitonially caused irreversible changes in the spleen such as fibrosis and leukocytes infiltration as a consequence of the administration route [68]. DODAB-induced focal necrosis in the liver at day 180 was milder than that caused by DOC/AMB, which was multifocal both at day 11 and day 180. In the kidneys, the formulation based on DODAB bilayer disks preserved the integrity of tubules and glomeruli in contrast to the serious damage caused by DOC/AMB. The majority of the toxic effects observed for the DODAB/AMB formulation were due to the DODAB carrier used at 10 mg/mL, i.e., at a rather high concentration so that substantial reduction of DODAB dose was required [68]. This led us to another DODAB/AMB formulation based on coverage of AMB aggregates with DODAB cationic bilayers that had a high drug-to-lipid molar ratio [63]. A tiny amount of the cationic lipid covered all drug particles with a lipid bilayer or, bilayer plus additional layers of biocompatible polymers virtually solving the toxicity problem associated with high doses of the cationic lipid [7,9,63]. A similar strategy was also used to formulate the fungicidal cationic drug miconazole [69] so that drug aggregates were covered by the anionic dihexadecyl-phosphate bilayers [26,70]. Alternatively, it was also possible to solubilize miconazole in the cationic DODAB discs with the disadvantages of the high doses of the toxic DODAB [71].

Besides the self-assembled cationic bilayers of the DODAB lipid [53,54,55,72], other nanomaterials often display antimicrobial activity by themselves as is the case of single-walled carbon nanotubes [73], or the metallic or metal oxide nanoparticles under UV irradiation that generate the reactive oxygen or nitrogen species, which kill not only microorganisms but also other cells and organisms of ecological importance [74,75,76]. These antimicrobial nanomaterials are discussed in the next sections.

## 3. BSA with Metals and Metal Oxide Nanoparticles

**Figure 4:**
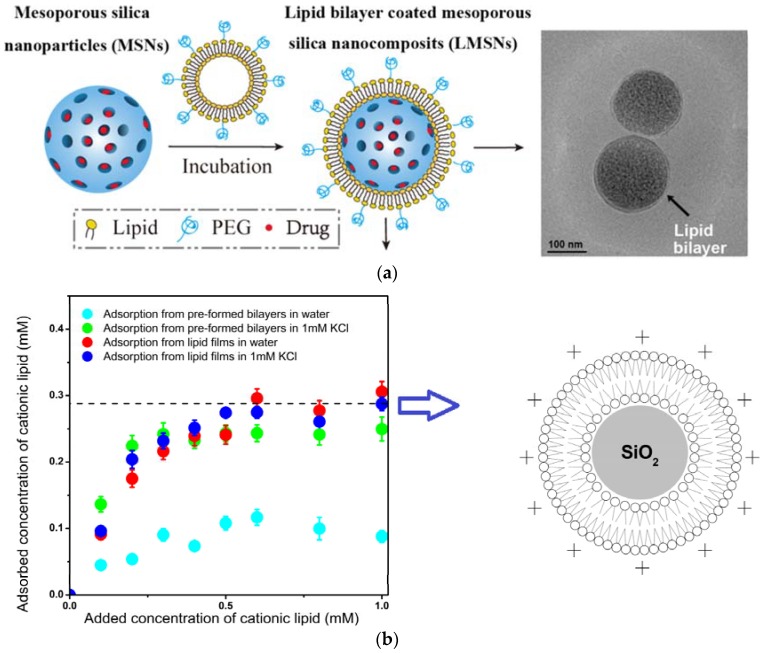
(**a**) Mesoporous silica particles as drug carriers with adsorbed phospholipid and pegylated-phospholipid bilayer as reproduced from [90]; reprinted from reference [90]. Copyright (2016) with permission of Elsevier. (**b**) Adsorption isotherms for the adsorption of cationic lipid on silica with bilayer deposition achieved for adsorption from lipid films at 1 mM KCl as reproduced from [87].

Commonly used inorganic or metallic nanoparticles such as silver (Ag), titanium dioxide (TiO_2_), silica (SiO_2_), zinc oxide (ZnO), and gold (Au) [77,78] have been found to exhibit bioactive properties [79] besides being useful in a variety of sectors such as energy, sensing, electronics, optics, ceramics, packaging, paints, agriculture, textiles, cosmetics, personal care products, pharmaceutics, biotechnology, drug delivery, imaging, etc. [77]. However, these NPs are nondegradable and persist in the aquatic environment affecting aquatic organisms such as prokaryotes, unicellular eukaryotes, and ciliates [77,80,81]; their steadily increasing use and release into the environment, especially into aquatic systems, inevitably raises the need of further research on the still unknown impact on aquatic ecosystems. In the mid-1990s, research on the toxicity of man-made, airborne nanoparticles towards pulmonary and other in vitro mammalian cell lines led to the conclusion that the ability of particles to generate reactive oxygen (ROS) and nitrogen radical species at or near their surface led to the oxidative stress in host cells as a central mechanism in their cytotoxicity [82]. Eventually, disguise of the inorganic particles and their toxic surfaces by coverage with biomimetic Trojan horses may become useful to develop biomimetic inorganic nanoparticles for delivery of antimicrobials [1,3,10,13,17,18,83,84,85,86,87,88]. The optimization of phospholipid bilayer adsorption on silica to obtain bilayer covered silica nanoparticles was achieved from a quantitative study of adsorption over a range of experimental conditions and used to reconstitute receptor-ligand recognition [83,89]. Recently, the optimization of silica coverage with cationic single bilayers of cationic lipid was also achieved by controlling ionic strength and the type of cationic lipid aggregates interacting with the silica particles [87]. Figure 4a illustrates the phospholipid bilayer coverage of mesoporous silica loaded with drug as reproduced from Han and coworkers [90]. Figure 4b shows some adsorption isotherms for optimization of cationic lipid bilayer coverage on silica obtained from DODAB films as compared with the insufficient coverage obtained from preformed bilayers [87]. 

Bioactive metal oxide nanoparticles as the zinc oxide ones (ZnO NPs) exhibit attractive antibacterial properties due to increased specific surface area corresponding to their reduced particle size. This increases the generation of reactive oxygen species (ROS) such as hydrogen peroxide, hydroxyl radicals, and peroxides with the increase in total surface area on the nanoparticles. ZnO bioactivity is related to this generation of ROS that damages the cell wall, increases the membrane permeability, dissipates the proton motive force, leads to uptake of toxic dissolved zinc ions, mitochondria weakness, intracellular outflow, and eventual inhibition of cell growth with cell death [76]. ZnO incorporation into packaging materials can cause interaction with foodborne pathogens, thereby releasing NPs onto food surface where they come in contact with food-borne bacteria and cause the bacterial death and/or inhibition [76]. Bioactivity for ZnO NPs encompasses bacteria, fungi, algae, and plants [75]. ZnO nanoparticles in its nanoscale form showed toxicity towards a wide range of microorganisms. In addition, using suitable surfactants or capping agents for surface modification, the desired physical properties such as size, shape and structure became available. Chitosan [76], carboxymethylcellulose [91], gelatin [92], agar [93], and starch [94] have been used to tune the surface properties of ZnO nanoparticles. Gelatin/ZnO NPs nanocomposite films aiming at food preservation showed antibacterial activity against both Gram-positive and Gram-negative foodborne pathogenic bacteria [92]. Similarly, polysaccharide-capped silver nanoparticles inhibited biofilm formation and eliminated multi- drug-resistant bacteria by disrupting bacterial cytoskeleton with reduced cytotoxicity towards mammalian cells [95]. The metal oxide nanoparticles can be prepared through both ex-situ and in-situ techniques [76].

Among the polysaccharides the versatile chitosan is the second most abundant biopolymer which is cationic at low pH, biodegradable, nontoxic, biocompatible; a partially deacetylated polymer of acetyl glucosamine obtained through alkaline deacetylation of chitin. Due to the presence of amino (–NH_2_) and hydroxyl (–OH) groups in the chitosan molecule, it acts as a cationic polymer over a range of low pH values [42]. Chitosan can easily interact with negatively charged surface/polyanions to form complexes and gels and was able to tune the properties of ZnO nanoparticles reducing crystallite size and enhancing the bioactivity of the composite nanoparticles [76]. 

## 4. BSA with Antibiotics

**Figure 5:**
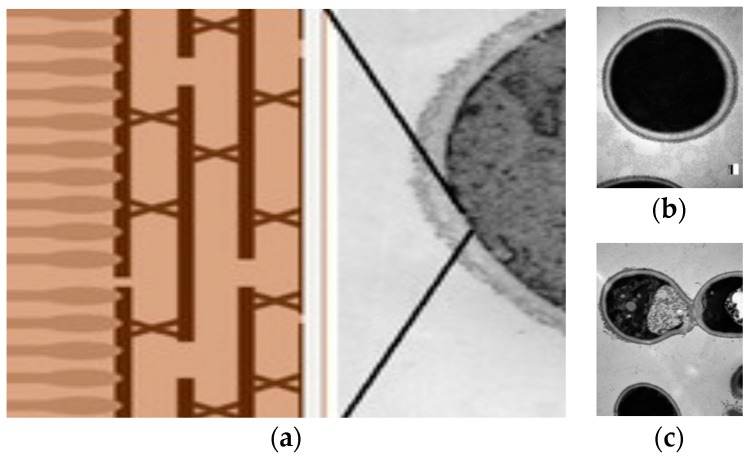
(**a**) Schematic molecular structure of yeast cell wall where the inner skeleton shows cross-linked polysaccharides and chitin parallel to the cell surface with an outer dense brush of glycoproteins [104]. (**b**) Transmission electron micrographs (TEM) of *Candida albicans* control. (**c**) TEM of *Candida albicans* damaged by a cationic antimicrobial polymer showing the burst of the cell membrane [106]. Adapted with permission from [106]. Copyright (2012) American Chemical Society.

Self-assembled antimicrobial nanomaterials can also spontaneously form from active antimicrobial molecules as are the antibiotics, carrier components and targeting moieties. They should be nanosized (10–100 nm), able to penetrate various tissues and even cells, stable in vivo for a sufficiently long time and should not elicit any toxicity. They should release the active antimicrobial component(s) upon contact with target microorganisms; and, at last, the components of the carrier should be easily removed or biodegraded from the body when the therapeutic function is completed [96,97]. Antibiotic therapy is often linked with low bioavailability, poor penetration to bacterial infection sites, the side effects of antibiotics, the antibiotic resistance properties of bacteria and/or quick antibiotic degradation in vivo [98]. Antibiotics encapsulation by appropriate carriers made up of a biodegradable polymer advantageously have been replacing the administration of antibiotics in their “free” form. Polymeric particles provide protection to antibiotics against environmental deactivation and alter antibiotic pharmacokinetics and biodistribution eventually overcoming tissue and cellular barriers [96,97]. For the optimal activity of a given antibiotic against intracellular bacteria, intracellular penetration and retention are required [99]. Antibiotics vary greatly regarding their ability to penetrate cells, as some, like macrolides, are efficiently taken up by cells and others, like aminoglycosides and vancomycin, are not [100]. The rapid uptake of the β-lactams into cells does not imply that intracellular accumulation occurs [101]. Basic aminoglycosides localize in the lysosomes where they are inactivated by the acidity [101,102]. Clarithromycin has difficult access to the phagosome, where mycobacteria reside, limiting its efficacy against mycobacteria in human macrophages [103]. Furthermore, the self-assembled nanomaterials exert their antimicrobial action differently depending on the microorganism being a fungus or a bacterium. The cationic antimicrobial polymers generally attack more easily the fungus than the cationic DODAB bilayers or the cationic nanoparticles do because they are able to insert in the fungus brush glycoproteins layer penetrating their outer wall firstly and then interacting with the fungus cell membrane causing lysis [41,104,105,106]. *Candida albicans* and *Cryptococcus neoformans* have thicker cell walls (∼280 and ∼200 nm, respectively) than Gram-positive bacteria (∼20–80 nm) [106,107,108]. Furthermore, the cell wall of *Candida albicans* is considerably less negatively charged than that of *Cryptococcus neoformans* (ζ-potential = −4 and ∼−30 mV, respectively) [108]. As a result, cationic nanoparticles or vesicles have difficulty in adhering to the *Candida albicans* cell wall in order to act on membranes and cause disruption and lysis [105]. The antimicrobial polymers by themselves seem to perform better these tasks than the nanoparticles bearing the same polymer as the outer layer displaying stronger activities against *Candida albicans* [5,7,9,104,106], sometimes in absence of cytotoxicity against red blood cells [104,109]. Figure 5a shows some structural features of the thick fungus cell wall showing its outer brush of glycoproteins [104]. Electron micrographs of control on Figure 5b and damaged cells affected by a cationic antimicrobial polymer on Figure 5c were from reference [106]. The effect of the free antimicrobial cationic polymer on the morphology of the fungus cell included the burst of the cell membrane in comparison to the unaffected control (Figure 5) [106].

In the next section, the importance of inert or bioactive polymers and their combinations with antibiotics or other antimicrobials in effective BSA to yield a variety of nanomaterials shapes such as nanoparticles, films, coatings and hydrogels will be discussed. 

## 5. BSA with Polymers

**Figure 6:**
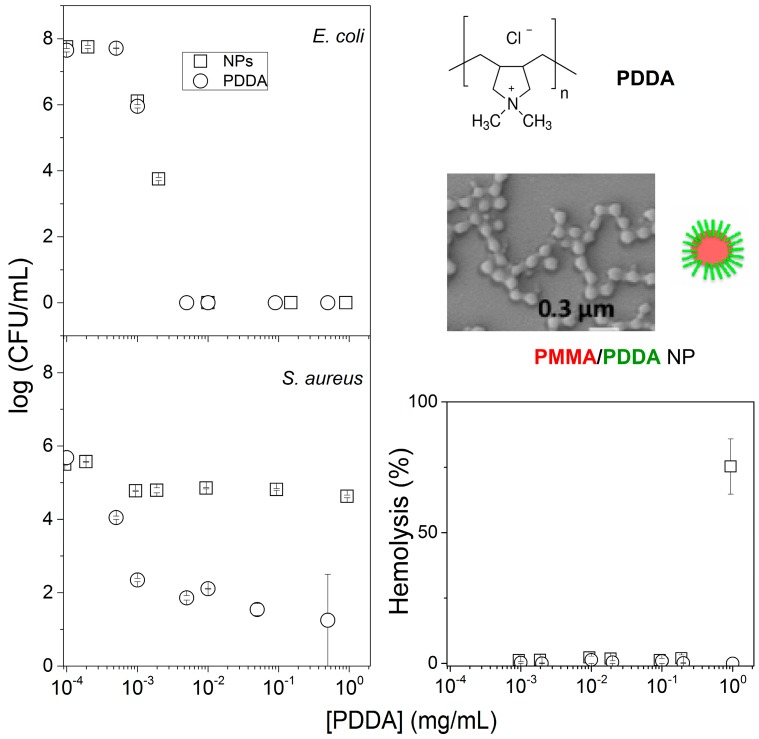
The compared bioactivity of a cationic antimicrobial polymer (PDDA) by itself and as the outer layer of PMMA/PDDA nanoparticles (NPs). On the left, colony forming unities (CFU) counting as a function of PDDA concentration against *Escherichia coli* and *Staphylococcus aureus.* On the right, PDDA chemical structure, scanning electron micrograph of PMMA/PDDA NPs and hemolytic activity of PDDA and PMMA/PDDA NPs as a function of PDDA concentration. Reproduced from [109].

As a first example of BSA with polymer, the cationic antimicrobial polymer PDDA or hybrid nanoparticles (NPs) of poly (methylmethacrylate) (PMMA) and PDDA were tested against three different cells: *E. coli*, *C. albicans* and red blood cells disclosing the superior activity of the cationic polymer by itself in comparison to the PMMA/PDDA NPs [109]. Figure 6 shows the core–shell structure of these PMMA/PDDA NPs obtained by the self-assembly of PDDA with PMMA. During polymerization of metylmethacrylate (MMA) to yield PMMA, the PDDA polymer present in the reaction mixture formed an outer layer (shell) around a core of PMMA. However, these cationic NPs when compared to the free PDDA polymer displayed inferior activity against Gram-positive bacteria and fungus; only against Gram-negative bacteria PDDA and PMMA/PDDA nanoparticles yielded microbicidal activity similar to the one of PDDA by itself [109]. Figure 6 shows the NPs core–shell structure, the comparison between the activity of the free PDDA polymer and the activity of the immobilized polymer forming a shell around a PMMA core of the PMMA/PDDA nanoparticles and the absence of lytic activity both for PDDA or PMMA/PDDA NPs.

The mechanism of killing may also involve the production of reactive oxygen species able to kill bacteria due to the deleterious effect of these species on vital biomolecules of the bacteria such as proteins, genetic material and lipids [110,111,112,113]. Given their importance as antimicrobial agents and their many applications in biomedical and dentistry devices, health care, food preservation, agriculture, catalysis, electronics, environment, renewable energy or textiles industry [114], the cationic antimicrobial polymers in a variety of non-covalent assemblies will be discussed in the next section. For example, antimicrobial polymeric coatings obtained from biocompatible polymeric particles adsorbing or mechanically immobilizing cationic antimicrobial polymers, cationic lipids or surfactants are versatile alternatives to prevent biofilm formation on the surfaces of a variety of materials thereby reducing infection [28,60,109,115].

Among the nanoparticles, encapsulation of aminoglycosides into polymeric biodegradable nanoparticles and polymer micelles allowed intracellular delivery. For example, poly(lactide-*co*-glycolide) acid (PLGA) nanoparticles improved the cellular uptake kinetics of gentamicin; PLGA nanoparticles effectively accumulated more than 10-fold gentamicin in phagocytic cells in comparison to the uptake of free gentamicin, with a predominant subcellular localization in the cytosol [116]. The uptake of gentamicin by phagocytic cells was also significantly enhanced by binding to other polymeric nanosystems, e.g., polymer micelles with poly(ethylene oxide)–poly(propylene oxide)–poly(ethylene oxide) triblock (PEO-b-PPO-b-PEO) shells and polyacrylate anion (PAA) cores (PAA−+Na-b-PEO-b-PPO-b-PEO-b-PAA−+Na), polymer micelles with PEO-b-PAA−+Na, polybutylcyanoacrylate (PBCA) nanoparticles [117,118,119]. By comparison with gentamicin solution, the binding to PBCA nanoparticles with dextran 70 as stabilizer produced a 5.34-fold increase in uptake by the macrophages at 0.5 h incubation and 26.74-, 8.03- and 7.36-fold increase in uptake by the hepatocytes upon 1, 12, and 24 h incubation; the cumulative uptake of encapsulated gentamicin increased with the incubation time [119]. The effective delivery of encapsulated gentamicin into cells augmented the therapeutic activity against intraphagosomal *S. aureus*, vacuolar Salmonella and intracytoplasmic *L. monocytogenes* [117,118,119]. The uptake route and rate of encapsulated gentamicin by macrophages are critically dependent on their physicochemical properties, nanomaterials, and preparation process. Amphiphilic block copolymers have been widely used in drug delivery; the biocompatibility of polyacrylamides and polyethylene glycol related systems has been useful in the delivery of antimicrobials and antibiotics. Biodegradable materials allow the release of antimicrobials from the matrix that can be a natural polymer such as gelatin or a synthetic polymer such as polyester, polyalkyl α-cyanoacrylate or polyamino acid [120]. These materials suffer hydrolysis and/or degradation by enzymes in the physiological environment, and eventually are absorbed or metabolized to water.

Several hydrophilic and biodegradable materials can be loaded with antibiotics via physical adsorption for further release in vivo. Some examples are cross-linked chitosan and amphicillin [121], octadecyl isocyanate-coated (poly(2-hydroxyethyl methacrylate)) and norfloxacin [122], carbopol and alginates with ciprofloxacin [123], polyurethane and tobramycin [124], or vancomycin [125], polypeptides and cefalozin and vancomycin [126], poly(trimethylene carbonate)-eroding systems and gentamicin/vancomycin [127], poly-(dl-lactic-*co*-glycolic acid) and ceftazidime [128] and (poly(hydroxybutyrate-polyhydroxyvalerate))/wollastonite with gentamicin [129]. Recently, cationized cyclodextrins and carboxymethylcellulose (CMC) assembled meropenem improving its chemical stability in a formulation that yielded nanoparticles [130]; rapid excretion by the kidneys and high instability in water solution due to opening of the β-lactam ring in meropenem could be circumvented thanks to the cyclodextrin (CD)/CMC NPs with 135 nm of mean diameter. The NPs increased drug bioavailability and stability in water media slowing down the β-lactam antibiotic hydrolysis at room temperature by nearly 30% while at 4 °C the hydrolysis was 63% slower [130]. 

Nanomaterials for antimicrobial drug delivery include liposomes, solid lipid nanoparticles, dendrimers, polymeric and inorganic nanoparticles [120,131]. Owing to their ultrasmall size, nanoparticle formulations have many advantages over traditional carriers. As for the contribution to prolonged action of active agent, they are capable of improving serum solubility of the drugs, prolonging the systemic circulation lifetime and releasing drugs in a sustained and controlled manner. Moreover, antibiotic-loaded nanoparticles can enter host cells through endocytosis and then release drug payloads to treat microbe-induced intracellular infections [119,131]. 

**Figure 7:**
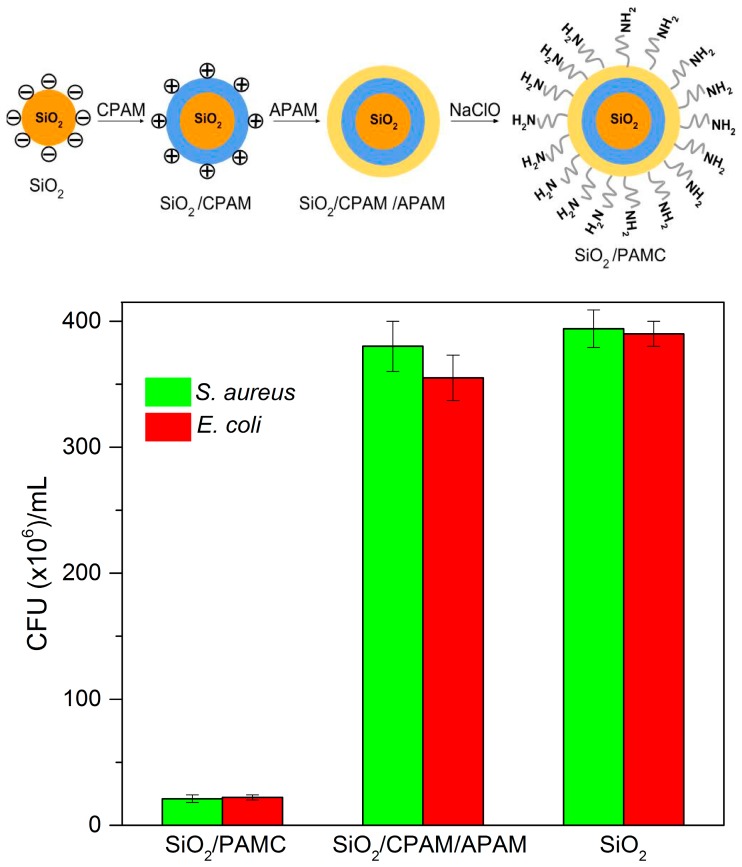
The microbicidal activity of cationic SiO_2_/anionic polyacrylamide (APAM) bleached nanoparticles (PAMC) from colony forming unities counting on agar plates. Adapted from [138] with permission from the Royal Society of Chemistry. CPAM: cationic polyacrylamide.

Cationic antimicrobial polymers are important for combating biomaterial-associated infections [1,42,132]. Polymers with quaternary ammonium [7,9,60,133], phosphonium [134,135], guanidine [136,137], *N*-halamine [138], isothiouronium [139] or DNase-mimicking polymer brushes that lyse bacterial DNA are able to prevent device-related infections deriving from biofilms [132]. For example, poly guanidine polymer displayed excellent activity against oral bacteria responsible for periodontal disease [136]. PDDA displayed excellent activity against *Candida albican*s in absence of toxicity against red blood cells [104]. The enzyme DNase cleaves bacterial DNA and prevents or disintegrates the established biofilms in growth medium. In another example, *N*-halamine-labeled core–shell silica–polyacrylamide from a layer-by-layer (LbL) electrostatic self-assembly process employed monodisperse silica nanoparticles for the negatively charged supporting core before adding a layer of cationic polyacrylamide (CPAM) and then anionic polyacrylamide (APAM) [138]. After treatment with bleach via sodium hypo chloride, the amide groups in the polymer shell were transformed into *N*-halamine-labeled silica–polyacrylamide core–shell nanoparticles (SiO_2_/PAMC) [138]. These core-shell NPs, described in reference [138] displayed bactericidal activity only for the cationic hybrid NPs with *N*-halamine moieties at the outer layer (Figure 7).

Hyperbranched polyamidoamines (h-PAMAM) functionalized with *N*-diazeniumdiolate nitric oxide (NO) donors and modified with polypropylene oxide (PO) were evaluated by studying their antibacterial activities and toxicity against common dental pathogens and human gingival fibroblast cells, respectively [140]. The combination of NO release and PO modification yielded h-PAMAM materials with efficient bactericidal action without eliciting unwarranted cytotoxicity [140]. Nitric oxide (NO) is an endogenously produced free radical that kills bacteria via nitrosative and oxidative stress [141]. NO loaded in and released from delivery scaffolds such as silica, gold, polymeric nanoparticles and dendrimers has often been employed as an efficacious antimicrobial [142,143,144].

**Figure 8:**
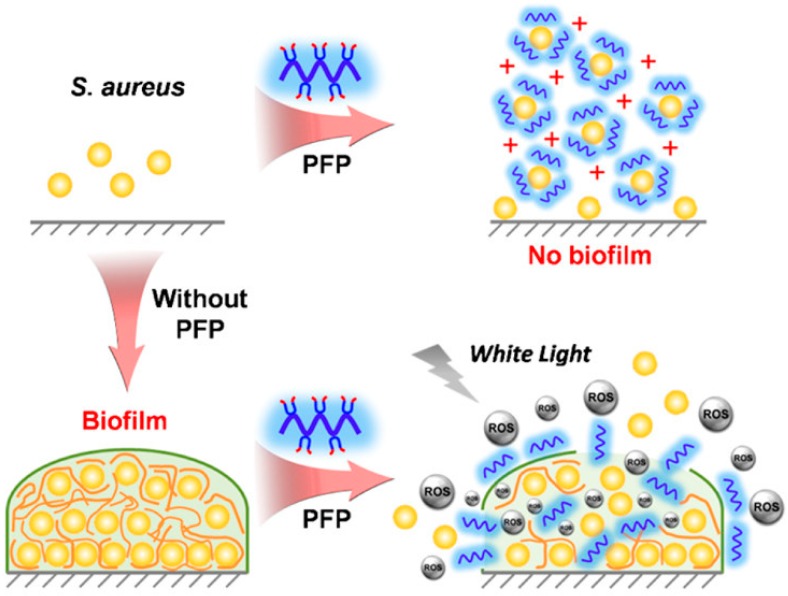
Scheme for the activity of water-soluble PFP (poly-{[(9,9-bis(6’-*N*,*N*,*N*-trimethylammonium)hexyl) fluorenylene phenylene]dibromide}) conjugated polymers against *S. aureus* planktonic cells as reproduced from reference [146]. Reprinted with permission from [146]. Copyright (2017) American Chemical Society.

Other interesting BSA based on cationic polymers were represented by the light-activated nanomaterials containing conjugated polyelectrolytes adsorbed onto colloidal particles or forming capsules [112,113,145]. Some water-soluble conjugated polymers such as poly-{[(9,9-bis(6’-*N*,*N*,*N*-trimethylammonium)hexyl) fluorenylene phenylene]dibromide} (PFP) with positively charged quaternary ammonium (QA) showed biocidal activity due to insertion of QA into the cell membrane and the ability to generate ROS by sensitizing oxygen molecules around, which not only inhibited biofilm formation but also eliminated mature and established biofilms thanks to the reactive oxygen species (ROS) produced by PFP under white light irradiation [146]. For these quaternary ammonium PFP the total antimicrobial activity reflected the combined light toxicity and dark toxicity. Figure 8 reproduced from [146] illustrates the process of PFP attack against *S. aureus* biofilms. PFP can penetrate the bacteria biofilm and continuously generate ROS under irradiation, resulting in biofilm disruption. Conjugated polymers are promising for the disruption of biofilms in biomedical and industrial applications.

Important aspects for the structure–activity relationship of biocompatible and cationic antimicrobial polymers such as poly (diallylammonium trifluoroacetate), poly (diallylmethylammonium trifluoroacetate) and poly (diallyldimethylammonium chloride) were recently elucidated [109,147]. Increasing the degree of hydrophobicity by methylation, increased activity against Gram-negative bacteria but did not affect Gram-positive bacteria or fungus. *Candida albicans* was very sensitive to PDDA and its derivatives. Comprehensive reviews on antimicrobial polymers disclosed their structural variety, activity and applications [148,149,150].

## 6. BSA with Peptides

**Figure 9:**
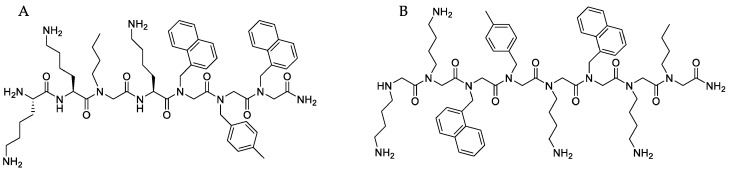
Chemical structures of synthetic peptidomimetic molecules such as a peptide–peptoid hybrid (**A**) and a peptoid (**B**); both highly active (MIC 2–4 µg/mL) for treating topical infections in dogs caused by *Staphylococcus pseudintermedius.* Reproduced from [152].

Important alternatives to antibiotics in order to overcome resistance are the antimicrobial peptides (AMPs) and peptoids [20,25,27,151]. The advantages of antimicrobial peptides are their broad-spectrum activity that encompasses the majority of Gram-positive and Gram-negative bacteria, their bactericidal and rapid action, low resistance, and low immunogenicity. However, the vast literature and clinical trials on AMPs did not result in systemic therapy and treatments [151]. AMPs are active selectively at micromolar concentrations and prone to have their chemical synthesis optimized despite their short half-life in vivo and high scaling-up costs of production making difficult the commercial uses [25,152]. De novo design of AMPs following a peptide-mimetic approach [153] yields peptoids [154,155,156], peptide–peptoid hybrids [157] and α-peptide/β-peptoids [158] with increased in vivo stability and resistance against proteolytic degradation. Most studies have been performed with short peptoids up to four residues.For example, a recent study described a peptide-peptoid hybrid, B1, and a peptoid, D2, that were highly active against *Staphylococcus pseudintermedius* (MIC 2–4 µg/mL) and exhibited potential as topical treatment against canine pyoderma [152]. Figure 9 illustrates the chemical structure of these interesting peptidomimetic molecules obtained by copying AMPs regarding their hydrophobicity and cationic character which gather positively charged and hydrophobic moieties for optimal activity as reproduced from [152].

In the food industry, consumers interest for natural products generated an increasing development of novel AMPs and AMPs derived lipopeptides that avoid the development of resistance in bacteria and fungi without acting on specific targets; further, the degradation of peptides used as food preservatives by proteolytic enzymes is desirable to avoid undesirable effect on gut microbiota [159,160]. However, the bacteriocin nisin still remains as the only antibacterial indeed used as food preservative [161] despite its restricted spectrum of activity [162]. Recently, the ultrashort peptide H-Orn-Orn-Trp-Trp-NH_2_ (O3TR) showed antifungal activity against several contaminants from food products and inhibited the growth of filamentous fungi and yeast species over a range of concentrations (12.5–50 μg/mL); the addition of lauric acid at the N-terminus of O3TR yielded the C12O3TR derivative, that was 2- to 8-fold more active than O3TR against every species with O3TR activity strongly reduced in salt solutions in contrast to the lauric acid peptide which kept its antifungal activity and resistance to proteolytic digestion [163]. There was a reduction in the random coil due to the conjugation with lauric acid, which increased the α-helical content and the hemolytic and the cytotoxic activity of O3TR, at the antifungal concentrations. After 7 days, O3TR inhibited yeast growth in beverages. This antifungal tetrapeptide thereby revealed its potency for novel food preservatives with good stability and low cytotoxicity.

Polymeric micelles with an average diameter smaller than 180 nm self-assembled from cholesterol-conjugated poly (ethylene glycol) (PEG) and transcriptional activator YGRKKRRQRRR peptide (TAT) (TAT-PEG-b-Chol); after loading with ciprofloxacin, they delivered this antibiotic across the blood–brain barrier (BBB) [164]. This approach may be important to treat brain infections since the endothelial cells of the brain tissue capillaries are connected by tight junctions and form a barrier that hampers the transport of antibiotics from the blood to the brain; for example, all β-lactam antibiotics penetrate poorly into the cerebro-spinal fluid (0.5–2.0%) when the BBB is normal. The TAT peptide peptide is part of the transcriptional activator TAT protein of the human immunodeficiency virus type-1 [164]. The assembly of TAT conjugated PEG-b-Chol into the polymeric micelles, which have a hydrophobic core of cholesterol for incorporation of antibiotics and a hydrophilic shell of PEG containing TAT molecules, enhanced the antibiotic uptake by the brain in a rat model [164]. In other instance, the careful design of the peptides improved the whole system. For example, three different peptides were obtained from *Pseudomonas aeruginosa*’s elastase (LasB) and combined with antimicrobial poly (ethylene imine); changing the number of anionic amino acids and cysteines per peptide affected particle preparation and stability so that increasing the charge and cross-linking potential of the peptides resulted in particles with better stability under physiological conditions and upon storage for responsive drug delivery [165].

Polyethylene glycol-stabilized lipid disks as carriers for amphiphilic antimicrobial peptides such as mellitin protected the peptide against tripsin degradation and assured its sustained antimicrobial activity [27]. In another self-assembled nanomaterial, the antimicrobial properties of cationic bilayer disks and antimicrobial peptides such as gramicidin were combined broadening the spectrum of activity of the formulation against bacteria [58,59] and also displaying activity against foodborne pathogens [11]. Interesting peptide assemblies combined as cationic nanofibers displayed the ability of penetrating cells disrupting their membranes and facilitating the entrance of additional drugs [166]. Similarly, cell-penetrating nanoparticles of self-assembled cationic antimicrobial peptides effectively killed the yeast *Cryptococcus neoformans* in a rabbit model of cryptococcal meningitis; importantly, unlike amphotericin B, they did not cause significant damage to the liver and kidney functions representing a promising alternative to amphotericin B for treating brain infections caused by *C. neoformans* [44]. 

Supramolecular assemblies based on peptides also require optimization of antimicrobial potency to achieve optimal activity in virtual absence of haemolytic or cytotoxic activity plus a desirable property of low cost for production when applied at the concentrations that exhibit antimicrobial potency. For example among the peptides, high stability and low cytotoxicity was achieved for an amidated tetrapeptide H-Orn-Orn-Trp-Trp-NH_2_ against food contaminants [163]. Positive net charge, hydrophobicity and amphipathicity are the key parameters of the antimicrobial activity of these peptides and many potent peptides are available based on these properties. However, the high cost of these peptides has been leading research towards shorter aminoacid residues and some of them display antimicrobial activity against several human pathogens as the antifungal tetrapeptide H-Orn-Orn-Trp-Trp-NH_2_ used to reduce fungal contamination of drinks and cereals. In addition to potency and safety, promising antimicrobials in food must present an interesting “low cost in use” property [154].

The need to understand the mechanisms that allow nanomaterials to overcome the bacterial envelopes has been focusing on cyclodextrins, nanoparticles, antimicrobial/cell-penetrating peptides and fusogenic liposomes [167]; cyclodextrins, for example, form water soluble cyclic oligosaccharides with a hydrophobic cavity that can enclose self-assembled hydrophobic drugs via noncovalent interactions improving delivery and biovailability of drugs targeted to mammalian cells [168]. The interaction between cationic peptides and the bacterial envelope is initially mediated by non-specific electrostatic interactions with the anionic lipopolysaccharides (LPS) and teichoic acids, in Gram-negative and -positive bacteria, respectively [169,170]. Anionic or neutral AMPs are possibly attracted to the divalent cations bound to LPS/teichoic acids [167] with additional help of hydrophobic interactions in the translocation of the peptides through the outer membrane in Gram-negative bacteria via a “self-promoted uptake” [171]. Neutral peptides with high activity against neutral membranes have hydrophobic peptide domains which provide a high membrane permeabilization efficiency [172]. AMPs already in clinical use include bacitracin, polymyxins (colistin and Polymyxin B), daptomycin, vancomycin and gramicidin though the need for proper AMPs formulation has been extensively recognized in the literature in order to improve bioactivity and sustained effect with time [173]

Self-assembled peptide based nanostructures such as nanotubes, nanofibers, nanoparticles, nanotapes and nanogels have been explored for biomedical applications; for example, stacking of peptide beta-sheets resulted in peptide nanotapes which eventually interacted with each other forming hydrogels [174]. Silver mineralization on self-assembled peptide nanofibers yielded efficient and long-term antimicrobial nanocomposites [175]. In biomedical device manufacturing the importance of preventing biomaterials-based infections led to several antifouling, contact-killing, and antimicrobials releasing surfaces [176]. However, surface attachment of peptides or even antimicrobial polymers does suffer from some disadvantages; the antimicrobial activity of the resulting coating may be strongly reduced compared to the activity of the peptide or antimicrobial polymer in free form [109,177,178,179]. Moreover, proteins, blood platelets, and dead bacteria may block the antimicrobial groups on the surface [176]. Since the antimicrobial activity is restricted to the surface of the implant, there is a lack of effect on bacteria in the tissue surrounding the implant so that contact-killing surfaces only eradicate bacteria that are in direct contact with the active surface and killing of any bacteria further away from the surface will depend on efficient phagocytosis and systemic or local antibiotics [176]. Nevertheless dual functionality as contact killing and release of dead bacteria was already reported for certain antimicrobial agents in combination with thermoresponsive polymers such as poly (*N*-isopropylacrylamide) (PNIPAAm); these polymers extend their chains at lower temperatures pushing out attached dead bacteria [148,180,181]. PNIPAAm combinations with antimicrobials were shown to be promising fouling-release materials that can release not only newly attached bacteria but also fully developed biofilms. Therefore, combinations of biocidal polymers and/or peptides with PNIPAAm should yield hybrid surfaces with dual capability: bacterial-killing and bacterial-release.

Other interesting approaches involved the controlled release of antibiofilm AMPs able to prevent experimental biomaterial-associated *Staphylococcus aureus* infection. The peptides were firstly incorporated in a polymer-lipid matrix which provided a constant release of 0.6% daily after an initial burst of about 50%; in a murine model this coating significantly reduced the number of culture positive implants and showed activity against multidrug resistant *S. aureus* [182]. Loading and release of AMPs could also be controlled from PEG-lipid disks [183], cationic bilayer fragments or nanodisks [11,58,59] or bilayer-covered polystyrene nanoparticles [10]. In the case of cationic lipid bilayers the combination of gramicidin D and the cationic lipid broadened the activity spectrum of gramicidin [59]. Whereas gramicidin D effectively kills *S. aureus*, the cationic lipid effectively kills *E. coli* [59]. 

For wound healing, peptide formulations have also displayed high therapeutic potential [24]. Peptides are small molecules that can be rationally designed to take advantage of their self-assembly in water solution for bottom-up construction of nanostructures such as spheres, cylinders, tubes, fibers and functional hydrogels; they represent promising compounds inherently biodegradable and biocompatible scaffolds with additional applications in cell therapies involving signaling, proliferation, and differentiation [184]. Peptide-based functional gels and membranes resemble the natural extracellular matrix and can be tailored to increase their interaction with cells and tissues [184]. For example stem cells were homogeneously encapsulated within three dimensional peptide hydrogels in a suitable formulation for cell delivery [185]. Recently, an excellent review on AMPs as therapeutic agents and promising delivery vectors appeared in the literature [186]. 

AMPs can also act synergistically with surface-active agents such as cetylpyridinium chloride [187] and DODAB [39] or antibiotics such as tigecycline, moxifloxacin, piperacillin–tazobactam, or meropenem [188] and others such as doxycycline [189], polymyxin E [190], clarithromycin [189,190,191], ampicillin, ceftriaxone, rifabutin, azithromycin and vancomycin [191]. The uptake of antibiotics in the presence of AMPs increased due to perturbation of the bacterial envelope and formation of pores by the peptide [192]. Hydrophobic antibiotics in the biological membranes can facilitate peptide entry [189]. The conjugation of a classical antibiotic to AMPs can also increase antibiotic activity and selectivity against bacteria [193]. 

Self-assembled peptide nanoparticles are also important in vaccine design [194,195]. Vaccination has been one of the most successful achievements in medical history and nanotechnology has significantly contributed to overcome the limited immunogenicity of the subunit-vaccines providing a variety of self-assembled adjuvants for antigen presentation [196,197,198]. Among these are the virus-like nanoparticles, the self-assembled peptide nanoparticles (SAPN), the bilayer discs or fragments and the biomimetic nanoparticles, all of them very promising [18,85,196,199]. SAPNs enhanced the immune response thanks to their repetitive display of antigens on the surface; their self-adjuvanticity advantageously eliminated further use of toxic adjuvants [194,199].

Other interesting antimicrobial nanomaterials are the nanostructured hydrogels loaded with antibiotics such as ciprofloxacin [200] or histatin-5 [201], the self-assembled nanofiber-based novel antibacterial ointment from antimicrobial peptides, bacitracin and gramicidin S [202], the soy lecithin-derived nanoliposomes [203] or the lipid-based liquid crystals with antimicrobial peptides [204], the model membranes with mellitin antimicrobial peptides and its analogues [205], ruthenium complexes/polypeptide self-assembled nanoparticles [206], antibiofilm peptides targeting a cellular stress response[207], and many others. In particular, biomedical applications for the bioactive peptide formulations in combating caries and pulpal infections[208] or for improving the outcome of implants and operative fractures care [209] or for enhancing the shelf-life of food are very important [210,211]. The strategies combining more than one antimicrobial are also important since they often improve the antimicrobial spectrum of activity and potency of the formulations [45]. Examples are the cospinning of silver nanoparticles with nisin in polymeric nanofibers [212], the co-encapsulation of the cationic peptide chrysophsin-1 and epirubicin in PEGylated liposomes able to circumvent multidrug resistance in HeLa cells [213], chitosan/temporin B nanoparticles with long-term antibacterial activity in vitro against clinical isolates of *Staphylococcus epidermidis* [214], and nisin Z combined with antibiotics in nanostructured lipid carriers enhancing antimicrobial activity[215]. Other major requirement in AMPs containing assemblies has been the sustained release for improving bioactivity such the delivery of lipopeptides from biodegradable polymeric carriers against oral pathogens [216]. The novel approaches for delivering AMPs have been recently and extensively reviewed in excellent articles [217,218,219,220,221,222,223,224,225]. 

## 7. Conclusions

In an attempt to categorize the self-assembled nanomaterials with antimicrobial activity, their shape was considered for summarizing the data in this review.

Table 1 summarizes some literature for the nanodisks. Table 2 shows self-assembled films, coatings, and hydrogels. Table 3 exemplifies some lipid-based nanomaterials such as vesicles, liposomes and lipid nanoparticles. Table 4 lists some self-assembled antimicrobial nanoparticles.

Although a search on self-assembled antimicrobial nanomaterials produces about 26,000 references, attempts to provide a synthetic and conclusive update are scarce. From 2014, about 19,000 references appeared on this subject. This review summarizes some relevant aspects of self-assembly and combinations between antimicrobials and major vehicles such as nanoparticles, bilayers, nanogels, polymers, and nanostructured lipid carriers emphasizing some of the most important applications of these self-assembled nanostructures in antimicrobial devices. The applications for the self-assembled antimicrobial nanomaterials span a broad range of strategic areas such as dentistry, medicine, drug delivery, pharmaceutical nanotechnology, vaccine design, food preservation, water treatment etc. Among the nanomaterials available important formulations involve combinations of two or more antimicrobials, toxic antimicrobials in biocompatible nanomaterials, and even toxic antimicrobials and toxic carriers. The exponential growth of options for antimicrobial nanomaterials have simple foundations based on physical intermolecular interactions giving support to the view that the coming together brings the strength for fighting the resistance of pathogens. From the effective BSA compositions examined in this review, it is possible to conclude that nanomaterials can protect liable active molecules, can be the active material themselves, can place antibiotics intracellularly for activity against intracellular pathogens, can hamper the formation of biofilms, and can sometimes accessorize the antimicrobial action by releasing dead pathogens from their contact-killing surfaces. 

## Figures and Tables

**Table 1:** Self-assembled nanodisks: composition and antimicrobial activity.

Composition	Antimicrobial Activity	Reference
DODAB/CMC/PDDA	*K. pneumoniae*, *MRSA*, *P. aeruginosa*	[5]
DODAB/Amphotericin B/CMC/PDDA	*C. albicans*	[7]
DODAB/rifampicin	*M. smegmatis*, *M. tuberculosis*	[8]
DODAB/CMC/PDDA	*P. aeruginosa*, *S. aureus*	[9]
PEG-stabilized lipid disks/mellitin	*E. coli*	[27]
DPPC/DODAB/Gr	*Not available*	[58]
DODAB/gramicidin D	*E. coli*, *S. aureus*	[59]
DODAB/gramicidin D	*E. coli*, *S. aureus*, *S. enterica*, *L. monocytogenes*	[11]
DODAB/amphotericin B	*C. albicans*	[57,62,68]
DODAB/CMC/PDDA	*C. albicans*	[104]

DODAB/CMC/PDDA: Dioctadecyldimethylammonium bromide nanodisks surrounded by carboxymethylcellulose and poly diallyl dimethylamonium chloride; MRSA: multidrug resistant *Staphylococcus aureus*.

**Table 2:** Self-assembled films (F), coatings (C), and hydrogels (H): composition and antimicrobial activity.

Composition	Antimicrobial Activity	Reference
F PMMA/DODAB; PMMA/CTAB	*E. coli*, *S. aureus*, *P. aeruginosa*	[28,29]
F nisin/pediocin/halloysite; nanoclay/nisin/pediocin/starch	*L. monocytogenes*, *C. perfringens*	[30]
F hydroxypropyl methyl cellulose/xyloglucan/gentamicin	*E. coli*, *S. aureus*	[36]
F essential oils, carvacol, thymol in halloysite NT/polyethylene	*E. coli*	[47]
F gelatin/ZnO/glycerol	*E. coli*, *L. monocytogenes*	[92]
F latex NPs/quaternized block copolymers	*P. aeruginosa*, *S. aureus*, *S. epidermidis*, *C. parapsilosis*	[115]
F copolymer of styrene, ethylene, butylene with DNase-like brushes	*E. coli*, *S. aureus*	[132]
F cellulose acetate/quaternized chitosan/anionic soy protein	*E. coli*, *S. aureus*	[133]
C poly hydroxy ethyl methacrylate/antimicrobial peptide	*S. aureus*, *P. aeruginosa*	[179]
C quaternary ammonium salt/poly (*N*-isopropylacrylamide)	*E. coli*	[180]
C PLGA-DPPC-DSPC-CHOL/AMP, doxycyclin	*S. aureus*, *MRSA*	[182]
C Phosphatidylserine coated silver NPs/allylamine plasma polymer film	*S. aureus*, *P. aeruginosa*, *S. epidermidis*	[226,227]
H cationic peptide with β-sheets forming fibers	*S. aureus*	[35]
H hydrophobic tripeptide/ciprofloxacin	*E. coli*, *S. aureus*, *K. pneumoniae*	[200]
H hydroxypropyl methyl cellulose/histatin-5	*C. albicans*	[201]

PMMA/DODAB: polymethylmethacrylate/dioctadecyl dimethyl ammonium bromide; PMMA/CTAB: polymethylmethacrylate/cetyltrimethylammonium bromide; NT: nanotubes; NPs: nanoparticles; PLGA-DPPC-DSPC-CHOL/AMP: Poly lactide-*co*-glycolide acid-dipalmytoilphosphatidylcholine-distearoylphosphatidylcholine-cholesterol/antimicrobial peptide.

**Table 3:** Self-assembled lipid-based nanomaterials: composition and antimicrobial activity.

Composition	Antimicrobial Activity	Reference
DODAB vesicles/rifampicin	*M. smegmatis*, *M. tuberculosis*	[8]
DODAB vesicles	*E. coli*, *C. albicans*	[53,54,55,73]
DODAB/DPPC vesicles	*Not available*	[58]
DODAB/Gramicidin D vesicles	*E. coli*, *S.aureus*	[59]
Clarithromycin/afloxacin/ethambutol/ phosphatidylcholine, dicetylphosphate, cholesterol	*M. avium*	[103]
Cubic glycerol/monooleate/water or hexagonal glycerol/monooleate/water/oleic acid and antimicrobial peptides	*MRSA*	[204]
Nanostructured lipid carriers/nisin Z/novobiocin	*S. aureus*, *S. epidermidis*	[215]

**Table 4:** Self-assembled nanoparticles: composition and antimicrobial activity.

Composition	Antimicrobial Activity	Reference
Clarithromycin/DODAB/CMC	*M. abscessus*	[6]
Amphotericin B/DODAB/CMC/PDDA	*C. albicans*	[7]
PSS/DODAB/gramicidin D	*E. coli*, *S. aureus*	[10]
Mesoporous silica/silver/antibiotic	*E. coli*, *K. pneumoniae*	[14]
Porphirin photosensitizer/cholesterol/anti-tuberculosis pro-drug	*M. fortuitum*	[15]
TAT amphiphilic peptide with hydrophobic cholesterol core	*MRSA*, *S. aureus*, *C. albicans*, *B. subtilis*, *C. neoformans*	[19]
TAT amphiphilic peptide with palmitic acid core	*MRSA*, *S. aureus*, *B. subtilis*, *E. coli*, *P. aeruginosa*	[20]
Amphotericin B/polyglutamic acid	*C. albicans*	[21]
Cholesterol-conjugated cationic G3R6TAT peptides	*C. neoformans*	[44]
PMMA/DODAB; PMMA/CTAB	*S. aureus*, *P. aeruginosa*	[60]
Silver/carboxymethyl tamarind polysaccaride	*E. coli*, *S. typhimurium*, *B. subtilis*	[95]
PLGA-PLH-PEG/vancomycin	*S. aureus*	[102]
PMMA/PDDA	*E. coli*, *S. aureus*, *C. albicans*	[109]
PLGA/hydrophobic gentamicin	*S. aureus*, *L. monocytogenes*	[116]
Triblock copolymer PEO-b-PPO-b-PEO/gentamicin	*S. typhimurium*, *L. monocytogenes*	[117,118]
*N*-halamine-silica-polyacrylamide	*S. aureus*, *E.coli*	[138]
Polyisothioronium methylstyrene crosslinked by ethylene glycol dimethacrylate	*P. aeruginosa*, *E. coli*, *S. aureus*, *L. innocua*	[139]
Hyperbranched poliamidoamine/*N*-diazoniumdiolate nitric oxide donors	Gram-negative and -positive periodontal and cariogenic bacteria	[140]
Block copolymers/gold/*N*-diazoniumdiolate NO donors	*P. aeruginosa*	[144]
TAT-PEG-b-cholesterol/ciprofloxacin	*E. coli*	[164]
Peptide polymer consisting of valine and lysine residues	*Streptococcus* and several others	[171]
Carbon/gold/nisin	*E. coli*, *P. aeruginosa*	[211]
Poly-d-lactide/chitosan/temporin B peptide	*S. epidermidis*	[214]

PSS: polystyrene sulfate; TAT: transcriptional activator YGRKKRRQRRR peptide; PLGA-PLH-PEG: poly(d,l-lactic-*co*-glycolic acid)-b-poly(l-histidine)-b-poly(ethylene glycol).
